# When in Doubt, Cut It Out: Biopsy as Key in Diagnosing Cryptococcal Soft Tissue Infection

**DOI:** 10.7759/cureus.21111

**Published:** 2022-01-11

**Authors:** Teresa Bernardes, Lorena Ostilla, Amara Fazal, Niloofar Nasseri-Nik, Christian Otrakji, Ghassan Haddad, Jorge Murillo

**Affiliations:** 1 Department of Medicine, South Miami Hospital, Baptist Health of South Florida, Miami, USA; 2 Department of Pathology, South Miami Hospital, Baptist Health of South Florida, Miami, USA

**Keywords:** skin infection, cryptococcal infection, atypical skin lesions, biopsy, soft tissue infection, cryptococcus

## Abstract

Soft tissue infection is an uncommon presentation of *Cryptococcus* in the absence of immunosuppression. Most infected patients present with pneumonia or meningitis, often with signs of disseminated disease, which may be fatal. We present a case of an 81-year-old mildly immunocompromised woman with multiple comorbidities, who presented with an extensive soft tissue infection on her right medial thigh. Superficial skin culture grew vancomycin-resistant *Enterococcus*; however, both initial and subsequent antibacterial therapies failed to resolve the infection. Subsequent biopsy revealed abundant yeasts, and mucicarmine staining confirmed *Cryptococcus* infection in a patient with no evidence of disseminated disease. Wound debridement and fluconazole for six months resulted in complete resolution of the lesion. In this report, we emphasize the need for tissue biopsy and microbial cultures in diagnosing patients with atypical skin and soft tissue infections who do not respond to appropriate antibiotics.

## Introduction

*Cryptococcus* is an encapsulated yeast that is ubiquitous in the environment, commonly found in fruits, vegetables, soil, and pigeon droppings [1]. Transmission to humans usually occurs via inhalation of airborne fungi conidia and hyphae fragments. Immunocompromised hosts are particularly vulnerable to life-threatening opportunistic infections such as cryptococcal meningitis and pneumonia [2-4]. Up to 10-15% of patients with disseminated cryptococcosis develop skin and soft tissue manifestations, whereas isolated skin and soft tissue cryptococcal infections are exceedingly rare, usually as a result of direct inoculation in both immunocompetent and immunocompromised patients [5-7]. Primary cutaneous cryptococcosis is a diagnosis of exclusion requiring evidence of local cryptococcal infection in the absence of clinical or mycological dissemination [5]. We present a case of subacute skin and soft tissue infection not responding to antibiotic therapy that required biopsy and special stains to demonstrate a locally invasive cryptococcal infection.

## Case presentation

An 81-year-old morbidly obese woman from Puerto Rico, living in Florida for 20 years, was admitted to the hospital with right thigh cellulitis that failed to improve after a seven-day course of oral clindamycin.

Her past medical history was significant for hypertension, rheumatoid arthritis on low-dose prednisone (2mg daily), chronic lower extremity lymphedema, atrial fibrillation on chronic oral anticoagulation, stage 3 chronic kidney disease, mild intermittent asthma, and obstructive sleep apnea. She underwent partial colectomy 17 years prior for localized colon cancer. Three months prior to presentation, she had been successfully treated for *Enterococcus faecalis* bloodstream infection without evidence of endocarditis.

**Figure 1:**
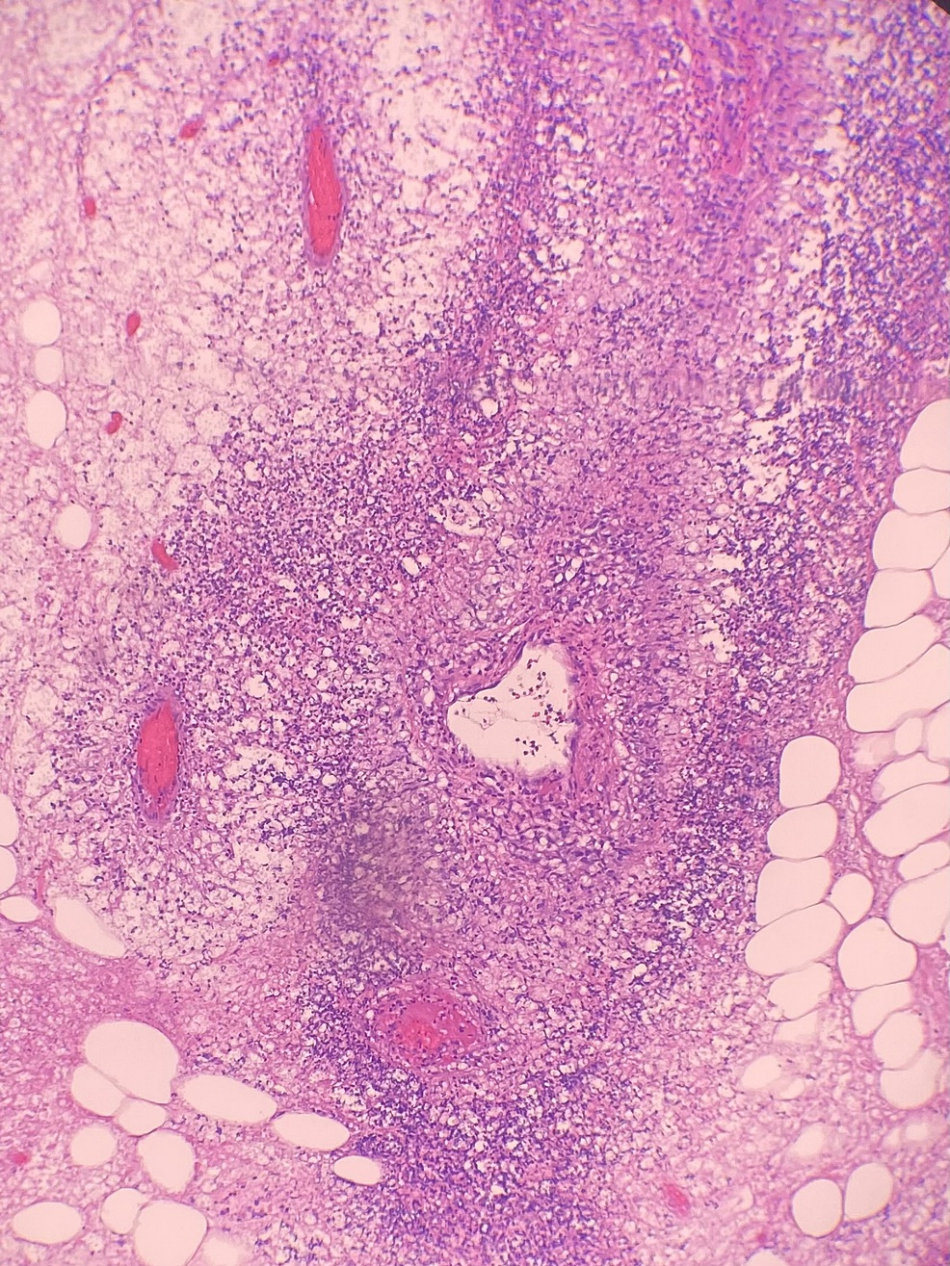
Light microscopy showing areas of necrotizing inflammation and vasculitis.

**Figure 2:**
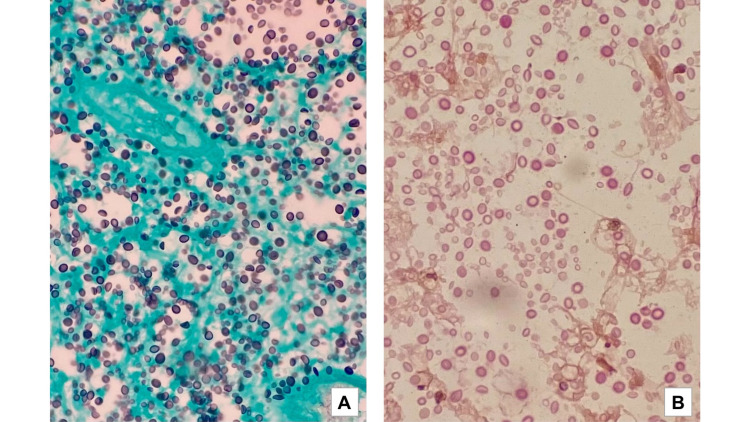
Light microscopy showing cryptococcal organisms, with characteristic peripheral capsular halo and budding forms, interspersed with mononuclear infiltrate, and enlarged adipocytes, suggesting marked inflammation and necrosis. Fungal elements stained with Gomori's methenamine silver stain (A) and mucicarmine-positive capsules (B), consistent with Cryptococcus infection.

Physical examination of the lower extremity revealed an extensive area of induration of the right inner thigh, with foci of superficial ulceration and skin necrosis. Computed tomography (CT) of the right lower extremity revealed thickening of the skin and subcutaneous tissue. A superficial culture of the wound grew vancomycin-resistant *Enterococci*, and the patient was started on linezolid 600mg orally every 12 hours, with no clinical response. Due to the atypical appearance of the skin lesion and lack of response to treatment, biopsy of the skin and soft tissue was performed, and the tissue was cultured for pyogenic organisms, acid-fast bacteria, and fungi. Histological examination of the tissue sample revealed necrotizing inflammation and vasculitis (Figure 1). Spherical encapsulated yeasts were highlighted by Gomori's methenamine silver (GMS) stain, and their cryptococcal nature was confirmed by mucicarmine staining of the capsules (Figures 2A, 2B). Subcutaneous fat and skin cultures were negative. Upon further questioning, the patient reported that she used to have daily contact with pigeons (a known carrier of *Cryptococcus*) while working in San Juan, Puerto Rico.

The patient was treated with intravenous fluconazole 400mg daily. Further workup was initiated to assess for cryptococcal infection risk factors and seek evidence of disseminated disease. Computed tomography (CT) of the chest, abdomen, and pelvis without contrast were unremarkable, and magnetic resonance imaging (MRI) of the brain did not reveal evidence of granulomatous disease. HIV-RNA was not detected, and the serum cryptococcal antigen titer was 1:64.

**Figure 3:**
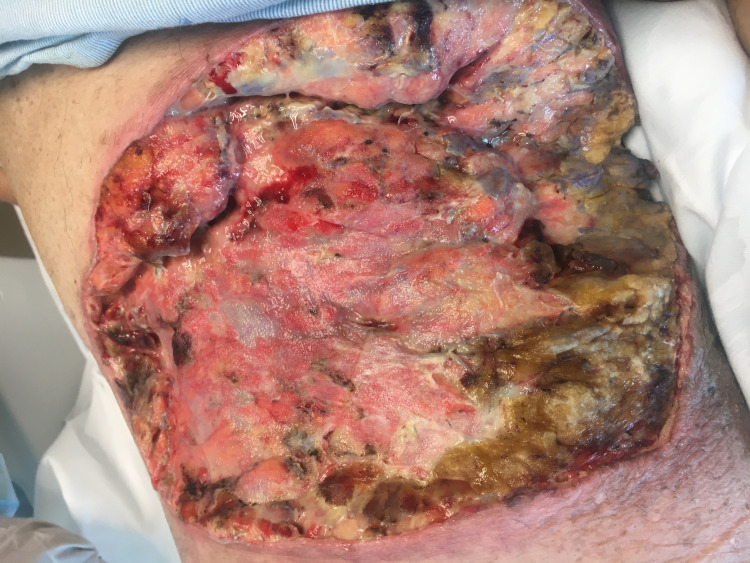
Right thigh wound after debridement.

The patient’s lesion was surgically debrided (Figure 3). She was subsequently discharged on oral fluconazole monotherapy for six months and referred for wound care as an outpatient. Full resolution of the lesion was observed at the six-month follow-up.

## Discussion

The skin is the third most common anatomical location of cryptococcal infection after the pulmonary and central nervous systems and is usually associated with disseminated infection in immunocompromised patients. Cases of primary skin infection have been reported. In a retrospective study including 108 patients with positive *Cryptococcus* skin cultures, 28 patients were described to have primary cutaneous cryptococcosis [5]. In a literature review including 21 immunocompetent patients with primary cryptococcal skin infection, patients did not have evidence of dissemination, had solitary lesions on exposed skin (phlegmon or whitlow), had a history of cutaneous injuries or exposure to bird guano, and showed *Cryptococcus neoformans* as well as *Cryptococcus gatii* isolated in cultures [8]. Moreover, other cases of directly inoculated skin lesions due to the emerging *Cryptococcus gatti *have been reported in immunocompetent patients [4,6,7]. *Cryptococcus gatti* is known to be more frequent in tropical and subtropical areas and can be found in Puerto Rico [7], where our patient was exposed to pigeons. Since our patient’s cultures resulted negative, we could not determine the *Cryptococcus* species.

Cryptococcal skin infection can manifest in a wide variety of ways. In immunocompromised patients, it can present as cellulitis or a soft tissue abscess, mimicking a bacterial infection [9,10]. Lesions can also be papular or maculopapular with an ulcerated center [10]. Occasionally, draining sinuses reflecting an abscess or an underlying bone lesion can be observed. When immunosuppression is severe, skin manifestations may simulate molluscum contagiosum, acne vulgaris, or malignancy [1,9-11]. Due to this diverse appearance, the physician must have a high index of suspicion and include primary cutaneous cryptococcosis in the differential diagnosis of a skin lesion that is not improving with empiric antibiotic therapy, regardless of their immunocompetent status. Patients with primary cutaneous cryptococcosis usually report living in rural areas, exposure to pigeons or eucalyptus, and occupation or hobbies that predisposed them to skin injury or a clear history of trauma [5,8]. Since there are no specific clinical manifestations, skin biopsy should be performed for special histopathological staining (GMS, mucicarmine, or alcian blue) and tissue cultures to establish the correct diagnosis [5,9]. We did not suspect *Cryptococcus* initially; however, since the infection worsened despite antibiotic therapy, we decided to obtain a biopsy of the lesion, which was key to the diagnosis of cryptococcal soft tissue infection and prompt initiation of therapy.

Because cryptococcal skin infections are usually a manifestation of disseminated disease in immunocompromised patients, a thorough workup for extracutaneous cryptococcosis and underlying immunosuppressive disorders is indicated. Our patient was elderly with mild chronic immunosuppression and a history of pigeon exposure, who did not have evidence of disseminated cryptococcal infection, supporting the diagnosis of primary cutaneous cryptococcosis. She had complete resolution after surgical debridement and fluconazole therapy for six months.

## Conclusions

Cryptococcal soft tissue infections are uncommon in immunocompetent or mildly immunosuppressed patients. Due to the lack of pathognomonic dermatologic features, the diagnosis may be overlooked in the absence of a high index of suspicion. With this case report, we emphasize the utility of tissue sampling along with special staining and cultures in diagnosing atypical skin lesions that fail to respond to antimicrobial therapy, regardless of the patient’s immunocompetent status.
